# Starch-Based Carbon Dots for Nitrite and Sulfite Detection

**DOI:** 10.3389/fchem.2021.782238

**Published:** 2021-11-05

**Authors:** Panyong Wang, Yan Zhang, Yulu Liu, Xinpei Pang, Pai Liu, Wen-Fei Dong, Qian Mei, Qing Qian, Li Li, Ruhong Yan

**Affiliations:** ^1^ School of Biomedical Engineering (Suzhou), Division of Life Sciences and Medicine, University of Science and Technology of China, Hefei, China; ^2^ The Affiliated Suzhou Science and Technology Town Hospital of Nanjing Medical University, Suzhou, China; ^3^ CAS Key Laboratory of Biomedical Diagnostics, Suzhou Institute of Biomedical Engineering and Technology, Chinese Academy of Science (CAS), Suzhou, China; ^4^ Jinan Guokeyigong Science and Technology Development Co., Ltd, Jinan, China

**Keywords:** carbon dots, on-off-on, nitrite and sulfite, starch, fluorescence detection

## Abstract

Nitrite and sulfite play important roles in human health and environmental science, so it is desired to develop a facile and efficient method to evaluate NO_2_^-^ and SO_3_^2-^ concentrations. In this article, the use of green alternatives with the potential of multi-functionality has been synthesized to detect nitrite and sulfite based on fluorescent probe. The carbon dots (CDs) with starch as only raw materials show fluorescence turn “on-off-on” response towards NO_2_^-^ and SO_3_^2-^ with the limits of detection of 0.425 and 0.243 μМ, respectively. Once nitrite was present in the solution, the fluorescence of CDs was quenched rapidly due to the charge transfer. When sulfite was introduced, the quenching fluorescence of CDs was effectively recovered because of the redox reaction between NO_2_^-^ and SO_3_^2-^, and thus providing a new way for NO_2_^-^ and SO_3_^2-^ detection. Owing to their excellent analytical characteristics and low cytotoxicity, the “on-off-on” sensor was successfully employed for intracellular bioimaging of NO_2_^-^ and SO_3_^2-^.

## Introduction

Nitrogen oxide is one of the primary pollutants from fuel combustion (Boningari and Smirniotis, 2016). The nitrite was thought to be inert end product of endogenous metabolism of nitric oxide (Lundberg et al., 2008). As food additives to inhibit the growth of microorganisms in cured and processed meats, excessive intake of nitrite ions with food or water can seriously endanger human health (Kalaycioglu and Erim, 2019). With potentially carcinogenic effects (Forman et al., 1985), nitrite can oxidize ferrous iron to trivalent iron to cause the formation of methemoglobin and has been listed as a highly toxic substance by The World Health Organization (Cockburn et al., 2013; Zhao W. et al., 2019). In addition, as a toxic air pollutant, sulfur dioxide is the main precursors of acid rain. Inhaled sulfur dioxide could be hydrated to produce it derivatives sulfite and bisulfite. Sulfite is considered as a restricted food additive in various food preservatives and excessive amounts of sulfite in food and drinking water have been major concerns for public health (Zhang et al., 2014; Wang et al., 2021b). It can cause harmful effects on tissue and has been found to be associated with asthma, hypotension, chronic obstructive pulmonary diseases, cardiovascular and gastrointestinal pain (Joseph et al., 2015; Pan, 2019; Asaithambi and Periasamy, 2020; Heaviside et al., 2021). In terms of the United States Food and Drug Administration (USFDA), the limit of sulfite residue in food is 10–100 ppm (Khan and Lively, 2020). Therefore, developing a rapid, highly selective and water-soluble probe to realize the sequential detection of nitrite and sulfite ions is of great importance.

In recent years, several analytical procedures including digital microfluidic platform, ion-exchange chromatography, ion-pair phase HPLC technique and capillary electrophoresis have been developed for the determination of these ions (Zuo and Chen, 2003; Iammarino et al., 2010; Della Betta et al., 2014; Gu et al., 2020; Zhang et al., 2021). However, these methods either require tedious sample preparation procedures, or are difficult to be widely used due to economic factors. Thus, a simple and inexpensive strategy to sense nitrite ions and sulfite ions with favorable sensitivity is highly desirable. There are many researches on sensing based on fluorescent nanocrystals. For example, lanthanide-doped fluoride nanocrystals are used for temperature sensing with ultrahigh relative sensitivity (Wang et al., 2021c; Wang et al., 2021d). However, their application may be hampered by complicated sample preparation procedure and sometimes the need for toxic raw materials. Carbon dots (CDs), on the other hand, can serve as a promising candidate in this field.

CDs, as these carbon-based fluorescent nanoparticles (typically less than 10 nm) has attracted the tremendous interest of researchers because of their unique properties such as low toxicity, excellent photostability, tunable emission spectra, easy surface functionalization, good biocompatibility and facile synthesis (Sun et al., 2006; Langer et al., 2021; Nazri et al., 2021). Because of these excellent properties, CDs have been applied in bioimaging, sensing, photocatalysis and drug delivery (Wang R. et al., 2018; Wang J. et al., 2019; Tosic et al., 2019; Yue et al., 2019; Jin et al., 2021). For instance, Qu et al. have synthesized bifunctional ibuprofen-based carbon dots for simultaneous bioimaging and anti-inflammatory (Qu et al., 2020). Jiao et la. have developed nitrogen-doped carbon dots for the ratiometric detection of sliver ions and glutathione (Jiao et al., 2019). Yarur et al. have demonstrated the synthesis of ratiometric fluorescence carbon dots for the detection of heavy metal ions with high selectivity and sensitivity (Yarur et al., 2019). As for detection nitrite ions in water, Zan et al. have reported green emission CDs for detection of nitrite ions and bioimaging (Zan et al., 2020). Another CDs synthesized by citric acid and amine were used for determining nitrite with a detection limit of 9.6 μg/L (Li et al., 2020). Chemical heteroatoms doping is an effective method to regulate the intrinsic properties of CDs. Jiang et al. have prepared polymer carbon dots doped with nitrogen and phosphorus to detect nitrite ions and the detection limit was as low as 0.55 μM(Jiang et al., 2019). Unlike nitrite sensors, the work of sulfite ions detected by fluorescence probes based on CDs have been rarely reported. The green fluorescence of upconversion nanoparticles was restored in the presence of sulfite or bisulfite and the limit of detection is 0.14 μM(Wang S. et al., 2018). Another method is the introduction of Cr (IV) into CDs and sulfite was successfully detected by the electron-exchange between Cr (IV) and CDs. The fluorescence of CDs was recovered when Cr (IV) was reduced by sulfite with the detection limitation 0.35 μM (Fang et al., 2017). Although fluorescent probes based on carbon dots have been developed to detect nitrite ions or sulfite ions, there are no reports using carbon dots for the sequential detection of nitrite ions and sulfite ions.

**FIGURE 1:**
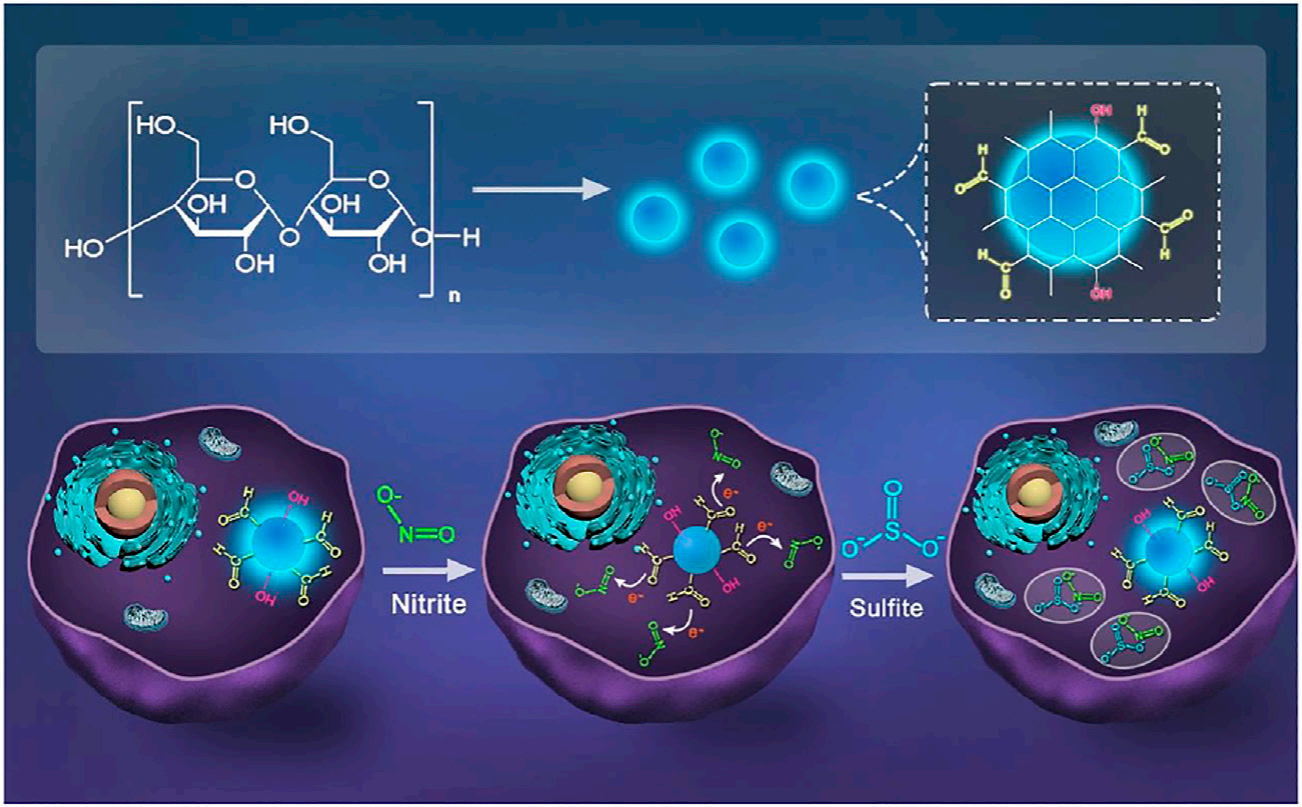
Scheme of synthesizing CDs and schematic illustration for the CDs detection of NO_2_^-^and SO_3_^2-^ in cells.

In this paper, we developed a CDs-based probe which can detect NO_2_^-^ and SO_3_^2-^ separately through a “on-off-on” mechanism. CDs were prepared using starch as raw material through one-step hydrothermal method, which is simple, environmentally friendly and suitable for large-scale production. The fluorescence intensity was quenched in the presence of nitrite ions and recovered with addition of sulfite derivatives (Figure 1). Taking advantage of fast response, stable fluorescence properties and favorable biocompatibility, CDs have been developed for the sensitive detection and imaging of nitrite ions and sulfite ions with the limits of detection of 0.425 and 0.243 μМ. In addition, the “on-off-on” detection systems for nitrite ions and sulfite display high sensitivity and selectivity, demonstrating the great potential of CDs in sensing, environmental science and food safety.

## Materials and Methods

### Materials

Starch, L-cysteine (Cys), glycine (Gly), urea, glucose (Glu), calcium chloride (CaCl_2_), sodium chloride (NaCl), copper chloride (CuCl_2_), potassium chloride (KCl), iron (III) chloride (FeCl3), glutathione (GSH), sodium sulfate (Na_2_SO_4_), sodium nitrate (NaNO_3_), sodium sulfite (Na_2_SO_3_), sodium phosphate (Na_3_PO_4_), sodium bisulfite (NaHSO_4_) and sodium nitrite (NaNO_2_) were purchased from Sinopharm Chemical Reagent Company (China). Dulbecco’s modified Eagle’s medium (DMEM) medium and fetal bovine serum (FBS) were purchased from HyClone (United States). WST assay kits were purchased from Energy Chemical (China). All regents were not processed or purified prior to use.

### Structure Characterization

Fluorescence spectra were recorded on a fluorescent spectrophotometer (F97Pro, China). UV-vis absorption spectra were recorded on a U-3010 spectrophotometer (Hitachi, Japan). Fluorescence lifetime measurements were carried out on photoluminescence Spectrometer (FLS 1000, United Kingdom). An AXIS ULTRA DLD spectrometer was used to detect X-ray photoelectron spectroscopy (XPS). Freeze dryers (Scientz-10N, CHINA) was used to obtain CDs solid powders. Transmission electron microscopy (TEM, JEOL Ltd, Japan) was used to characterize the morphology of the CDs. Nano ZS/ZEN3690 (Malvern, United Kingdom) was used to investigate the particle size distribution and surface potential of the CDs. Fourier transform infrared (FT-IR) spectra was acquired using an FT-IR spectrometer (Agilent Cary 660, United States).

### Synthesis of CDs

The synthesis of CDs was similar to our reported method (Wang et al., 2021a). Typically, 0.5 g starch was dissolved in 30 ml ultrapure water, stirred and ultrasonic vibrated for 10 min, and then heated at 200°C in a 100 ml stainless steel autoclave lined with polytetrafluoroethylene for 10 h. After the solution was cooled to room temperature, centrifuged at 10,000 rpm for 10 min to remove precipitate, filtered by a 0.22 μm filter membrane to further detach the aggregates and then dialyzed against pure water through a dialysis membrane (M_w_ = 1,000 Da) for 8 h. The product was lyophilized to obtain dark brown CDs and exhibited strong fluorescence under UV irradiation.

### NO_2_^-^ and SO_3_^2-^ Fluorescence Assay and Selectivity Studies

To detect of NO_2_^-^, different concentrations of NO_2_^-^ solutions (10 mM, final concentration 0–700 μM) were added systematically into 3 ml aqueous solutions of CDs (20 μL; the final concentration is 20 μg ml^-1^), then the sample was oscillated for 5 min at room temperature with a small oscillator at 1,000 rpm. Finally, the emission spectrum of the sample was measured by fluorescence spectrometer at the excitation wavelength of 360 nm. To verify detection selectivity of CDs toward NO_2_^-^, other ions solutions were examined in a similar way. For the assay of SO_3_^2-^, various concentrations of SO_3_^2-^ (10 mM, final concentration 0–700 μM) were obtained by diluting the stock solution with ultrapure water. The subsequent experimental procedure is consistent with the NO_2_^-^ detection process.

### Relative Fluorescence Quantum yields

The fluorescence quantum yield is the efficiency of converting absorbed photons into emitted photons (Grabolle et al., 2009). For the unknown sample relative fluorescence quantum yield, we can according to the known absorption and emission of relatively perfect quantum yield standard such as rhodamine 101, quinine sulfate and rhodamine 6G to obtain (Wurth et al., 2013). The QY of CDs was measured using quinine sulfate (55%) as standard (Olmsted, 1979) and was calculated using following equation:

$$QY_{s}=QY_{st}\left(A_{st}/A_{x}\right)\left({I_{x}/I}_{st}\right){\left({\eta_{s}/\eta }_{st}\right)}^{2}$$

Where A_st_ refers to the absorbance of the standard, A_x_ is the absorbance of the sample to be tested, I represent the emission intensity integral, η represents the refractive index of the solution. The subscript st represents the standard (quinine sulfate), and s represents the sample to be tested (CDs). For more reliable results and to minimize errors, A_s_ and A_st_ were less than 0.05.

### Cytotoxicity Assay

In briefly, HeLa cells were cultured in 0.4% penicillin streptomycin and 10% fetal bovine serum for 24 h in a 5% CO_2_ incubator at 37°C, and then the cells were diffused into 96-well plates (100 μL per well, 5,000 cells) and treated with CDs at different concentration (0–500 ug mL^-1^). After incubation for another 24 h, cytotoxicity of the CDs for HeLa cells was evaluated via a WST assay. The absorbance of each well was measured by a microplate reader at 450 nm after 4 h.

### Cell Fluorescence Imaging

HeLa cells were seeded on the coverslips in 6-well plates and incubated at 37°C under 5% CO_2_ in DMEM medium containing 10% FBS and 1% penicillin-streptomycin for 24 h. Subsequently, HeLa cells were treated CDs (200 μg ml^-1^) for a period of 24 h and washed three times with PBS for imaging. For the detection of NO_2_^-^ and SO_3_^2-^, these cells were incubated with 500 μM NO_2_^-^ for 0.5 h. In order to restore the intracellular fluorescence, SO_3_^2-^ (500 μM) was added and incubated for another 0.5 h. After washing the cells three times with PBS, the fluorescence images of the samples were observed using a confocal laser microscopy.

## Results and Discussion

### The Characterization of CDs

**FIGURE 2:**
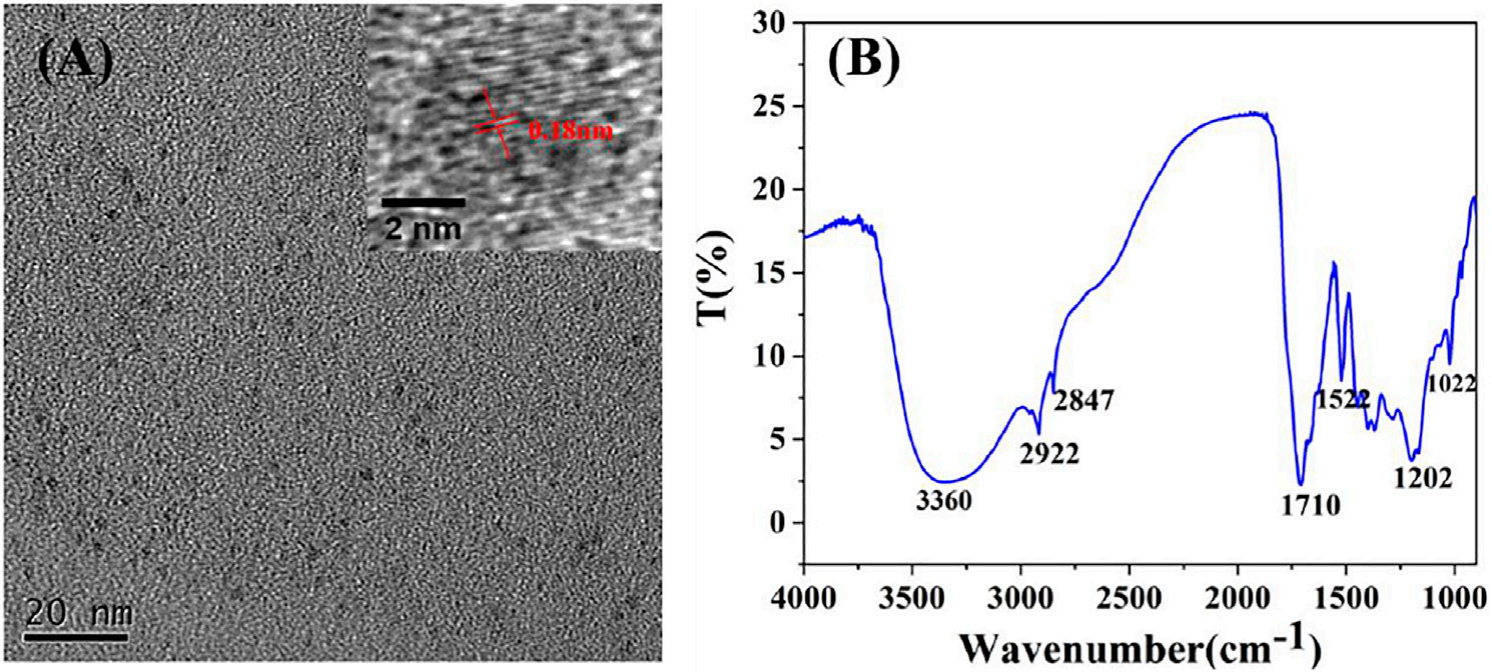
**(A)** TEM image and HRTEM of CDs **(B)** FT-IR spectrum of CDs sample.

The morphology, surface functional groups, structure and composition of the CDs were investigated by transmission electron microscope (TEM), Fourier transform infrared spectroscopy (FT-IR), Particle size analyzer (ZS nano 90) and X-ray photo-electric spectrometry (XPS). As illustrated in Figure 2A, the TEM image and the corresponding histogram of size distribution (Supplementary Figure S1) illustrated that CDs with the average particle size of 5.6 nm were uniformly dispersed and spherical shape, indicating they were water-soluble. High resolution TEM image (insert in Figure 2A) showed that the particles have a lattice structure and lattice constant is 0.18 nm. In the FT-IR spectrum of the CDs (Figure 2B), the broad peak at 3,360 cm^-1^is the telescopic vibration from O-H, the peak at 2,922 cm^-1^ corresponds to the stretching vibration peak of C-H and the peak at 1710 cm^-1^ comes from the stretching vibration of C=O (Ge et al., 2015; Li et al., 2015). The characteristic peak at 1,522 cm^-1^, 1,202 cm^-1^ and 1,022 cm^-1^ corresponds to the stretching vibration peak of C=C bond, C-C bond and C-OH bond (Zhi et al., 2018), indicating the existence of hydroxyl and various other moieties (such as C-H, C=O and C=C) in the CDs.

**FIGURE 3:**
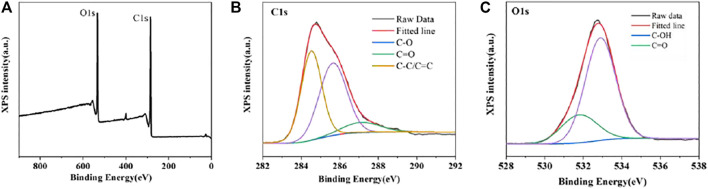
XPS spectra of CDs **(A)** Full-scan spectrum and high-resolution spectrum of C 1s **(B)** and O 1s **(C)**.

Then, XPS was performed to further identify the structural information of CDs. The XPS full scan spectrum in Figure 3A contains two distinct peaks at 284.8 (C 1s) and 532.8 eV (O 1s). Further, the major peaks at 284.5, 285.7 and 287.2 eV in the high-resolution C 1s spectrum are respectively the signal peaks of C-C/C=C, C-O and C=O groups. The O 1s XPS spectrum of CDs can be decomposed into peaks at 531.8 and 532.9 eV corresponded to the C=O and C-OH groups. These results further confirm that the presence of carboxyl and hydroxyl functional groups on surface of CDs, which is consistent with the results of the FT-IR spectrum.

**FIGURE 4:**
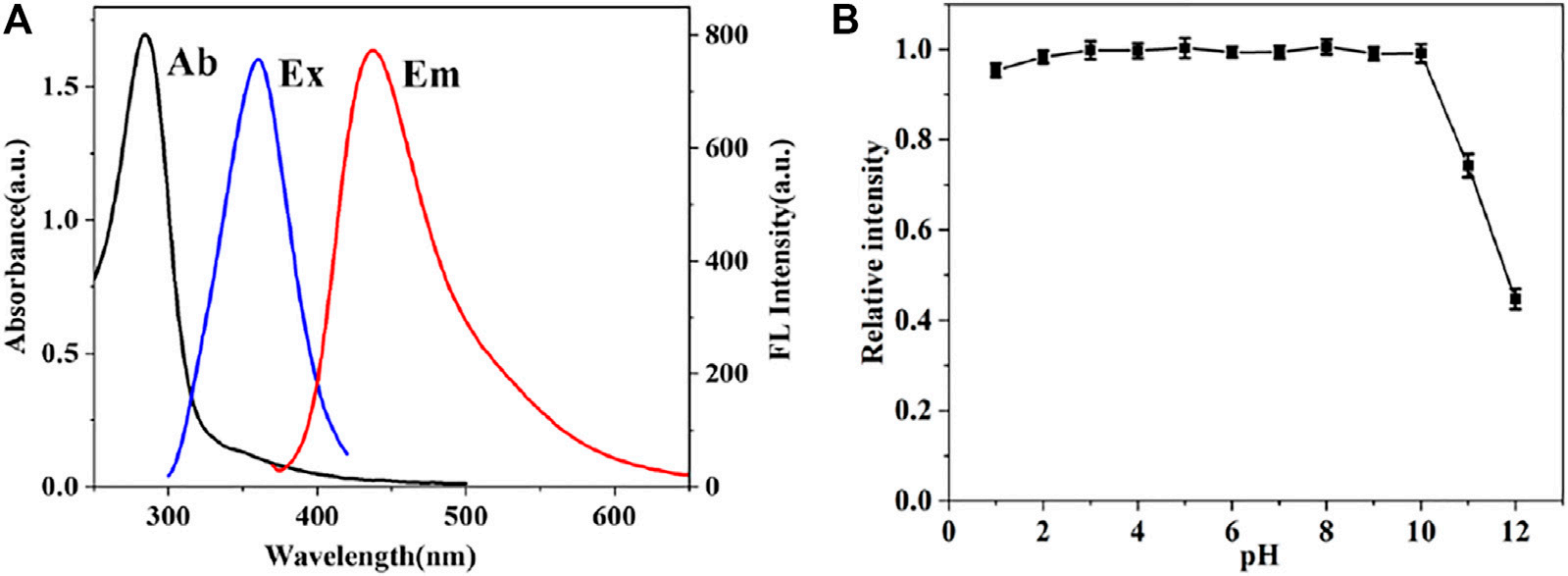
**(A)** UV-vis absorption spectrum (blank line), fluorescence excitation (blue line) and emission spectra (red line) of CDs **(B)** Influence of the pH on the fluorescence intensity of CDs.

To further investigate the optical properties of CDs, UV-vis absorption and fluorescence spectroscopy were also performed. Figure 4 exhibits an intense absorption peak at 284 nm, which is mainly originated from the π-π transition of C-C bond. Moreover, the maximum fluorescence emission intensity of CDs is located at 435 nm and excitation wavelength at 360 nm. In addition, CDs preserved stable fluorescence in solution with a wide range of pH values from 1 to 10. As demonstrated in Figure 4, the fluorescence intensity of CDs changed slightly by 5% under extreme acidic conditions, which may be due to a large number of hydroxyl groups on the surface of CDs. However, strong alkaline conditions can seriously affect the intensity of CDs. The NO_2_^-^ and SO_3_^2-^ detections in this work were all performed under natural conditions. Besides, the stability of the fluorescence intensity of CDs solution after storage for different time periods was also evaluated. The fluorescence intensity of the CDs solution decreased by only 13% during 6-days storage period, which indicate the good fluorescence stability of the CDs. And the relative quantum yield of CDs is 12.2% by using quinine sulfate as a reference. The robust fluorescence stability makes the CDs suitable for further bioimaging applications.

### Fluorescence and Selectivity Response of CDs Toward NO_2_^-^ and SO_3_^2-^

**FIGURE 5:**
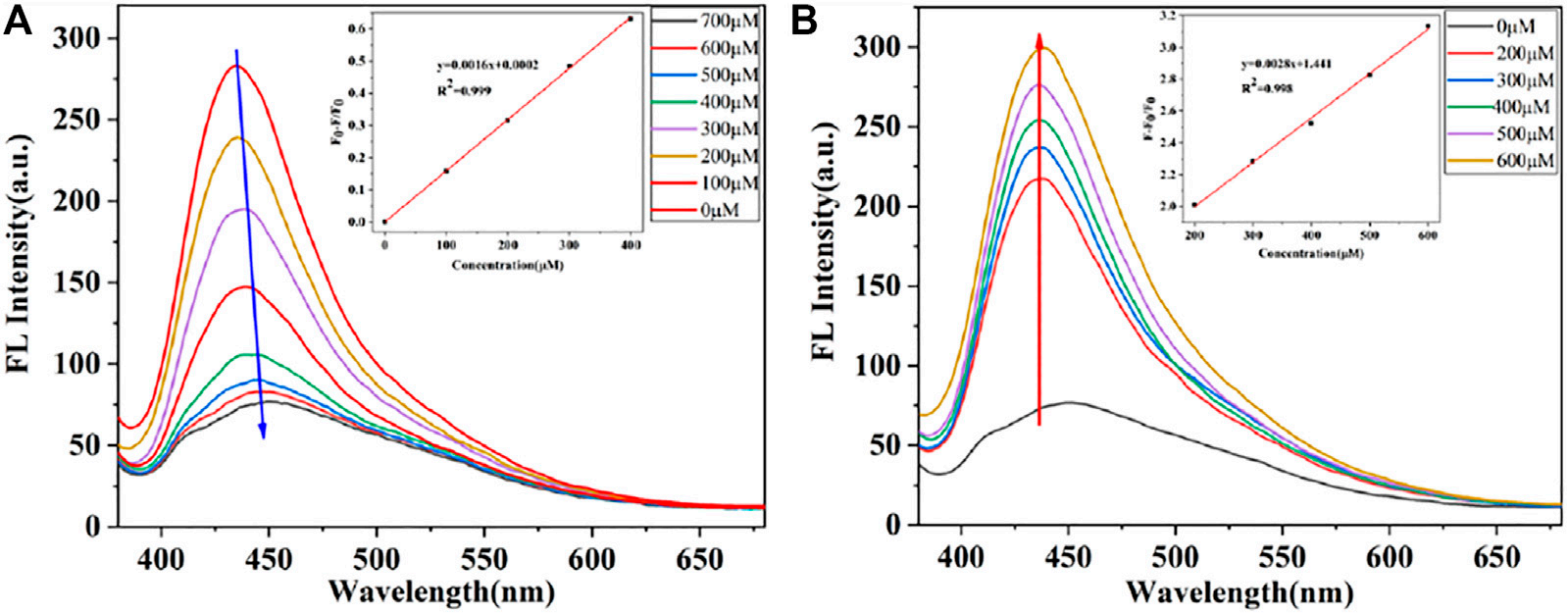
**(A)** Fluorescence spectra of CDs in the presence of different concentrations of NO_2_^-^. Insert: Linear relationship between (F_0_-F)/F_0_ and the concentration of NO_2_^-^ **(B)** Fluorescence recovery of the CDs with various SO_3_^2-^ concentrations. Insert: Fitted line between (F-F_0_)/F and the concentration of SO_3_^2-^.

The “on-off-on” fluorescent probes based on CDs were developed to detect NO_2_^-^ and SO_3_^2-^. As shown in Figure 5A, CDs have a specific binding ability with NO_2_^-^ and the emission fluorescence intensity of CDs at 435 nm was gradually quenched along with the increasing concentration of NO_2_^-^. Also, the fluorescence intensity of CDs was quenched over 60% after adding of NO_2_^-^ at the concentration of 400 μМ, and then, the downward trend slows down with the NO_2_^-^. Furthermore, it is worth to point out that there was an excellent linear relationship (*R* ^2^ = 0.999) between the fluorescence ratio (F_0_-F)/F_0_ and the concentration of NO_2_^-^, where F_0_ and F are the fluorescence intensities of the CDs in the absence and presence of NO_2_^-^. In addition, the limit detection of CDs for NO_2_^-^ was 0.425 μМ (LOD = 3σ/S, where σ is the standard deviation of the blank and s is the slope of the linear calibration plot). With the addition of SO_3_^2-^, the fluorescence intensity of CDs gradually recovered. As shown in Figure 5B, the emission intensities of this probe at 435 nm were recorded at 15 min after adding various concentration of SO_3_^2-^, which showed good linear relationship (*R* ^2^ = 0.998) between the fluorescence ratio (F-F_0_)/F_0_ and the concentration of NO_2_^-^ in the range of 200–600 μМ. The limit detection for SO_3_^2-^ was determined to be 0.243 μМ. The detection performance of CDs based “on-off-on” fluorescent sensor was comparable to previous reports (Table 1), articulating the availability and simplicity of the proposed sensing probe. Therefore, the results show that CDs can be considered as a good fluorescent probe for monitoring the concentration of NO_2_^-^ and SO_3_^2-^ with excellent sensitivity.

**TABLE 1:** Performance comparison of different fluorescence probes for the detection of SO_3_^2-^ and NO_2_^-^

Materials	Ions detected	Limitation (μМ)	Reference
Ammonium citrate (CDs)	SO_3_^2-^	0.35	Fang et al. (2017)
Gold nanoclusters	SO_3_^2-^	12	Sachdev et al. (2019)
NIR-SO2-TP	HSO_3_^2-^	1.06	Zhao et al. (2019c)
Corn	HSO_3_^2-^	0.5	Zhao et al. (2019a)
Fluorescein	SO_3_^2-^	1.74	Zhang et al. (2016)
Indole	SO_3_^2-^	0.57	Venkatachalam et al. (2020)
Ru-CHO	HSO_3_^2-^	0.52	Zhang et al. (2018)
Nicotinic acid, folic acid	NO_2_^-^	21.2	Gan et al. (2020)
Citric acid, phenylenediamine	NO_2_^-^	0.65	Jia et al. (2019)
Tris, urea	NO_2_^-^	13.5	Karali et al. (2018)
Sodium phytate, Na_2_SO_4_	NO_2_^-^	0.3	Wang et al. (2019b)
Starch	NO_2_^-^, SO_3_^2-^	0.425,0.243	This work

**FIGURE 6:**
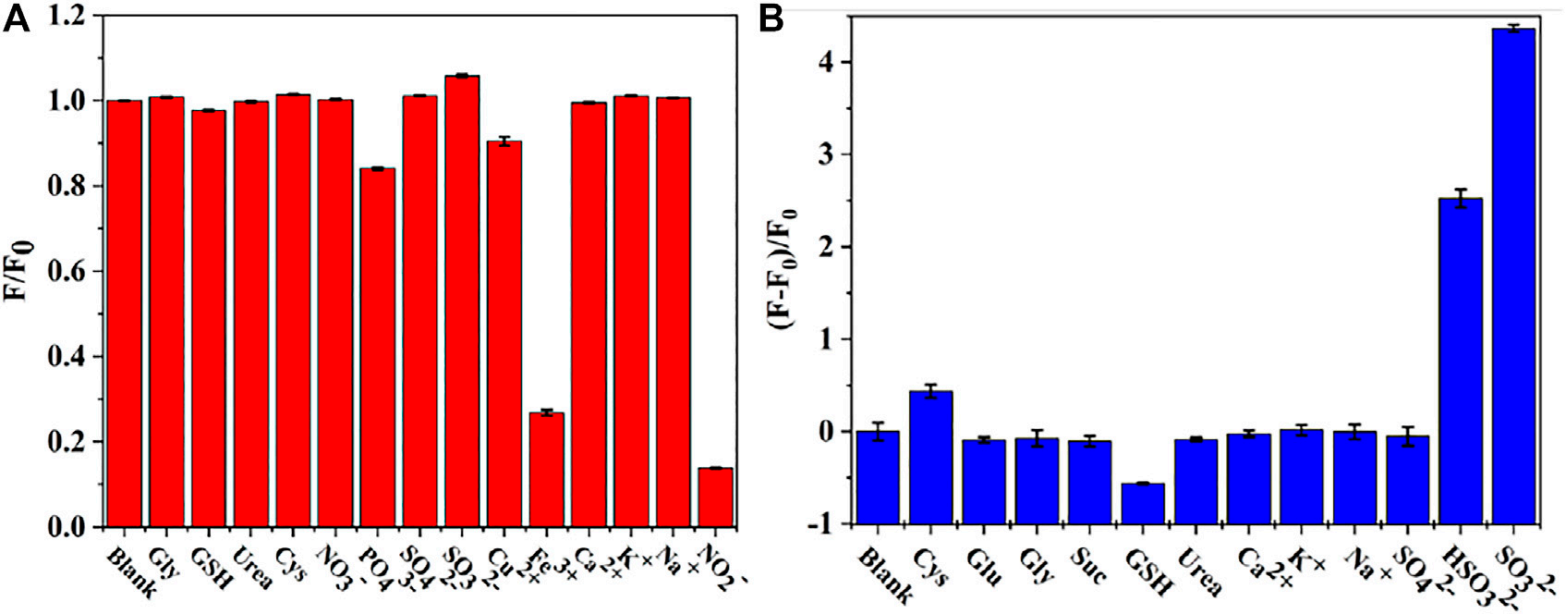
**(A)** Selective test of the assay toward NO_2_^-^ **(B)** Selectivity in the sensing response of CDs/NO_2_^-^ toward SO_3_^2-^.

In order to evaluate the selectivity in sensing response of CDs, various metal ions (Cu^2+^, Fe^3+^, Ca^2+^, K^+^, Na^+^), anions (NO_3_^-^, PO_4_^3-^, SO_4_^2-^, SO_3_^2-^) and organic molecules (Gly, GSH, Cys, Urea) were considered. Figure 6A displays the fluorescence intensities of CDs in the presence of NO_2_^-^ as compared to multiple interfering ions. The fluorescence intensity was reduced by 90% by NO_2_^-^ (500 μМ). Therefore, CDs show desirable selectivity for the detection of NO_2_^-^. Although, Fe^3+^ affect the fluorescence intensity of CDs. Fortunately, the concentration of Fe^3+^ in plasma is low (Supplementart Table 2) and the false signals can be effectively shield by triethanolamine. Thus, CDs have potential for directive and selective detection of NO_2_^-^ ions. Moreover, to evaluate the selectivity in sensing response of CDs to SO_3_^2-^, various metal ions (Ca^2+^, K^+^, Na^+^), anions (SO_4_^2-^, HSO_3_^2-^) and organic molecules (Gly, GSH, Glu, Cys, Urea, Suc) were investigated for their impact on the fluorescence intensity of CDs/NO_2_^-^. As illustrated in Figure 6B, the fluorescent responses and the corresponding luminescence variations of organic molecules and metal ions was negligible compared to the presence of SO_3_^2-^. The degree of fluorescence intensity recovered by HSO_3_^2-^ was equivalent to 57.8% fluorescence recovered by SO_3_^2-^. Therefore, the results confirmed that CDs had great potential for specifically detecting NO_2_^-^ and SO_3_^2-^.

### Mechanism of the Fluorescence Response of CDs to NO_2_^-^ and SO_3_^2-^

**FIGURE 7:**
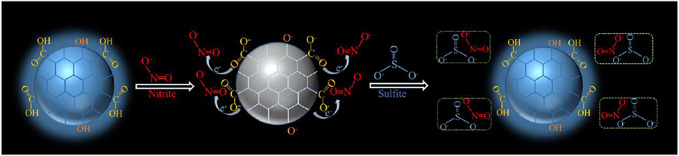
Schematic diagram of CDs with NO_2_^-^ and SO_3_^2-^.

To understand the fluorescence quenching mechanism of CDs, fluorescence lifetime decay, zeta potential, UV-vis absorption (Supplementary Figure S2) and electrochemical behaviors (Supplementary Figure S3) were investigated. After adding NO_2_^-^ and SO_3_^2-^, signals of UV-vis absorption were almost unchanged and these results were consistent with observation in the cyclic voltammogram (CV) after adding NO_2_^-^ and SO_3_^2-^ (Supplementary Figure S3). The CV of CDs just showed a reversible redox reaction. Moreover, the fluorescence lifetimes were respectively 1.8 ns (CDs only), 2.03 ns (in the presence of NO_2_^-^) and 2.55 ns (in the presence of NO_2_^-^ and SO_3_^2-^) in the Supplementary Figure S4, which displayed no obvious change and it is different from a dynamic fluorescence quenching mechanism, suggesting a static fluorescence quenching effect occurred. In detail, the zeta potential of CDs solution was measured as -36.85 mV, which indicates that the nucleus of CDs is positively charged and the surface is rich in anions. After the introduction of NO_2_^-^, it replaces the anions on the surface of CDs. And the strong electron absorption of NO_2_^-^ makes it difficult for the electrons in the CDs core to be excited, leading to fluorescence quenching. When SO_3_^2-^ was introduced, SO_3_^2-^ would reduce NO_2_^-^ and destroy the charge transfer between NO_2_^-^ and CDs, resulting in fluorescence recovery (Figure 7).

### Cytotoxicity Test and Intracellular Sensing

Before imaging, the cytotoxicity of CDs to HeLa cells was assessed using WST assay. Various concentrations of CDs (20, 50, 100, 200, 300, 400, 500 μg ml^-1^) were added to Hela cells and cell viability was observed more than 90% after incubating HeLa cells with CDs for 24 h (Supplementary Figure S4). Owing to their low cytotoxicity and excellent biocompatibility, the fluorescent probe was used to image NO_2_^-^ and SO_3_^2-^ in live cells.

**FIGURE 8:**
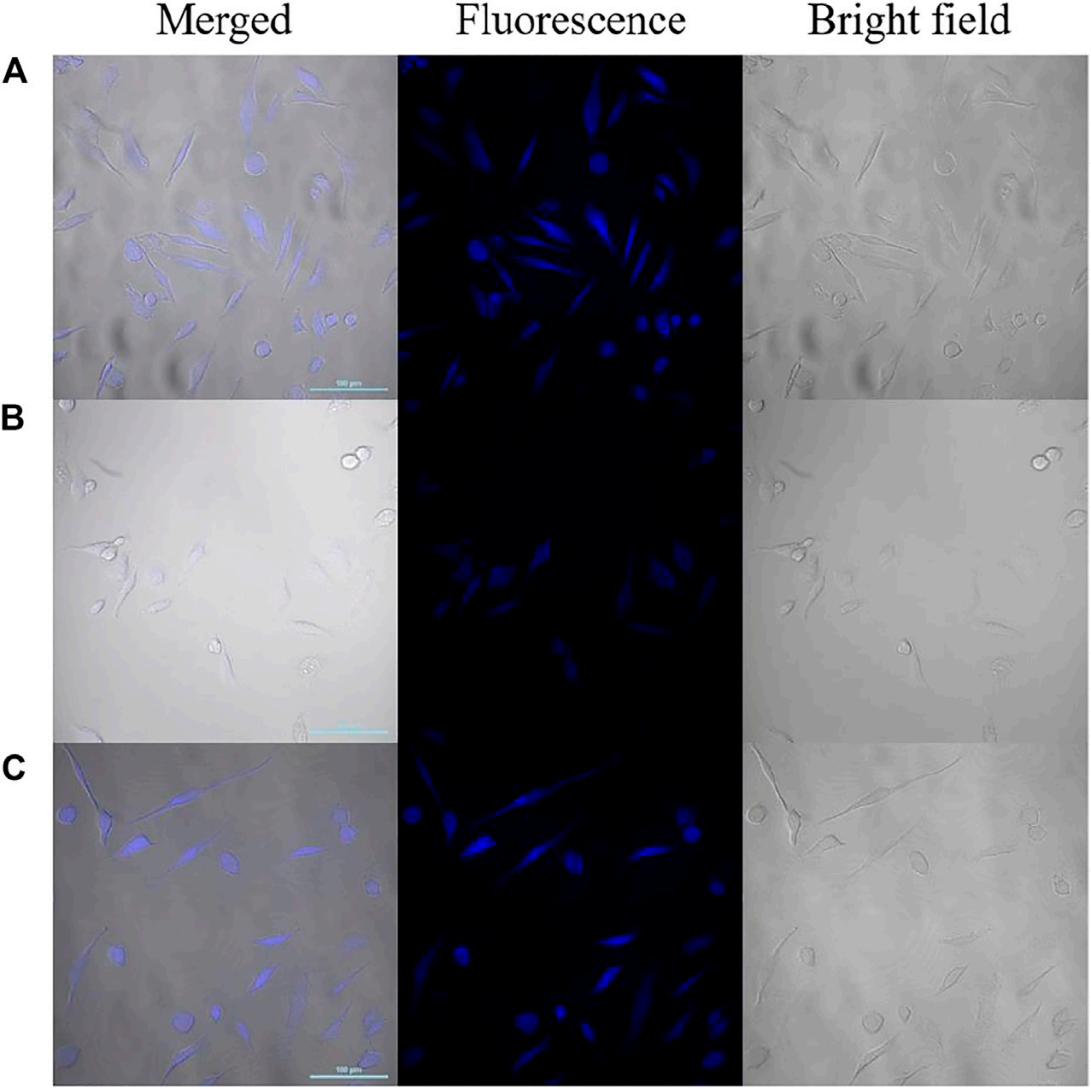
Confocal laser fluorescence images of HeLa cells incubated with CDs **(A)**, CDs/NO_2_^-^ **(B)** and CDs/NO_2_^-^/SO_3_^2-^ **(C)** (Left: the merged image; middle: the fluorescence image; right: the bright field image; scale bar: 100 μm).

The possibility of CDs to be as a label agent for fluorescent bioimaging of NO_2_^-^and SO_3_^2-^ was tested by a confocal laser microscopy. As shown in Figure 8, Hela cells treated with CDs solution (200 μg ml^-1^) exhibit blue fluorescence and retained normal morphology. Upon incubation with NO_2_^-^ for 20 min, the fluorescence in the living cells was significantly quenched. After adding SO_3_^2-^, the fluorescence recovered effectively. These results are consistent with the observed results in spectral experiments, which suggest that the fluorescent probe have the potential to detect NO_2_^-^ and SO_3_^2-^ in living cells.

## Conclusion

In summary, we have developed a highly sensitive and selective fluorescence probe for the detection nitrite ions and sulfite ions. The fluorescence of CDs was efficiently quenched by nitrite ions through a static quench mechanism, which was confirmed by the fluorescence lifetime. Because of the specific reactive response of CDs to nitrite ions and sulfite ions, the fluorescence of “on-off-on” sensor was quenched via nitrite ions and the weak fluorescence was enhanced upon addition of sulfite ions. Thus, the fluorescent probe can be used to detect nitrite ions and sulfite ions with convenience, high sensitivity and selectivity. Owing to low cytotoxicity and good biocompatibility, CDs have been used to image NO_2_^-^ and SO_3_^2-^ in HeLa cell. Therefore, this method may provide a new route for sensing nitrite and sulfite derivatives in environment and living cells.

## Data Availability

The original contributions presented in the study are included in the article/Supplementary Material, further inquiries can be directed to the corresponding authors.
