# Supplementary material for: Starch-Based Carbon Dots for Nitrite and Sulfite Detection
Source: Front Chem. 2021 Nov 5;9:782238. doi: 10.3389/fchem.2021.782238 (PMC8602874; doi:10.3389/fchem.2021.782238)
Supplement: Supplementary file 1 [file Table1.DOCX]

**Starch-based Carbon Dots for Nitrite and Sulfite Detection**

**Panyong Wang ^1,3^, Yan Zhang ^2,3^, Yulu Liu ^1,3^, Xinpei Pang ^1,3^, Pai Liu ^3^, Wen-Fei Dong ^3,4^, Qian Mei ^3,4^, Qing Qian ^3^*, Li Li ^3^*, and Ruhong Yan ^2^***

^1^ School of Biomedical Engineering (Suzhou), Division of Life Sciences and Medicine, University of Science and Technology of China, Hefei 230026, China;

^2^ The Affiliated Suzhou Science & Technology Town Hospital of Nanjing Medical University, Suzhou 215153, China;

^3^ CAS Key Laboratory of Biomedical Diagnostics, Suzhou Institute of Biomedical Engineering and Technology, Chinese Academy of Science (CAS), Suzhou 215163, China;

^4^ Jinan Guokeyigong Science and Technology Development Co., Ltd, Jinan 250103, China

***** Correspondence: yrhzl@hotmail.com (R. Yan), lil@sibet.ac.cn (L. Li).


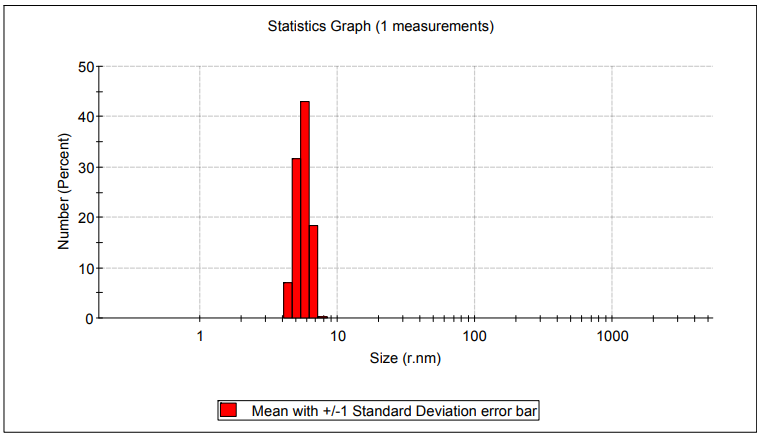


**Figure S1**. Particle size distribution of CDs.


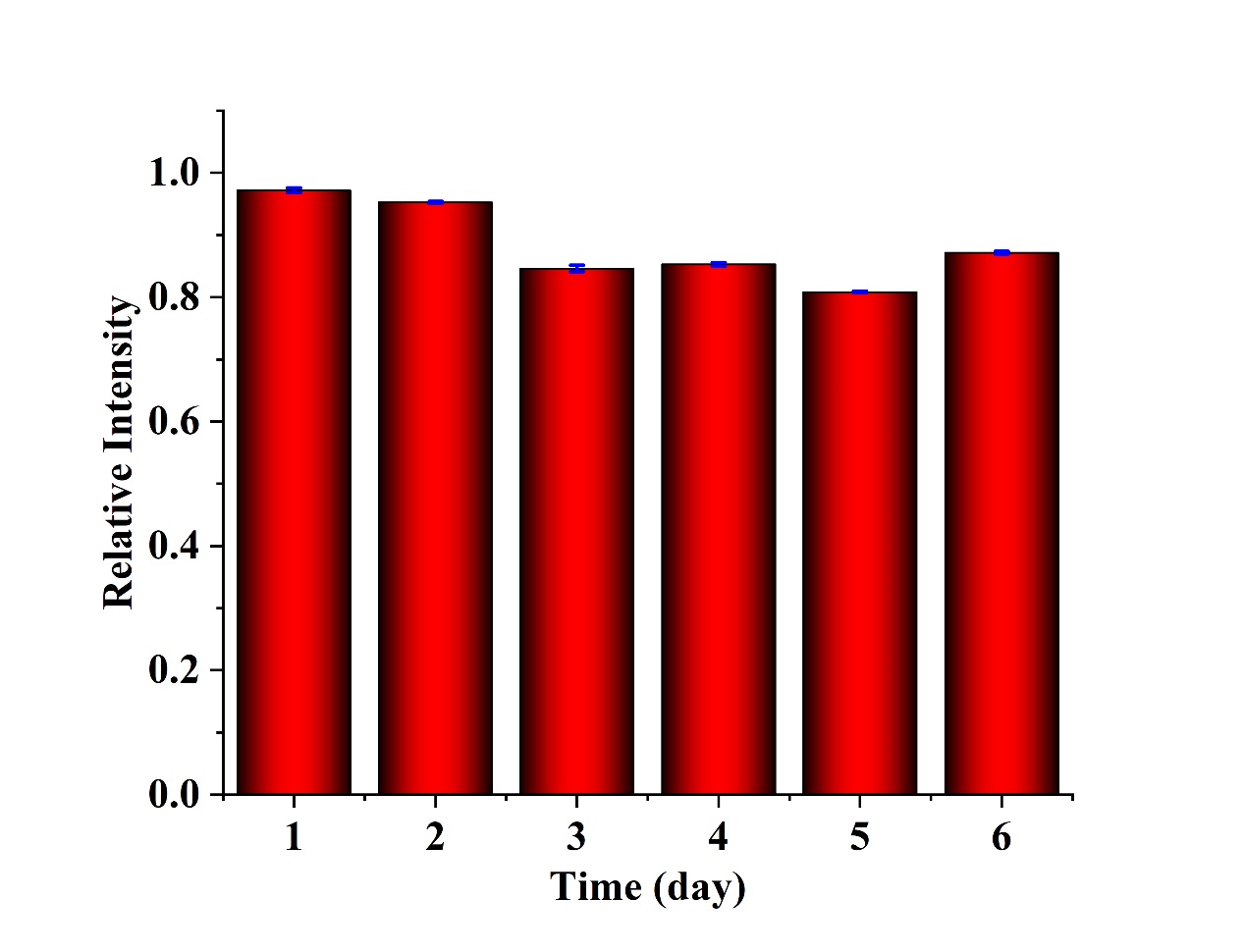


**Figure S2.** The fluorescence intensities of CDs solution after storage for different time periods.


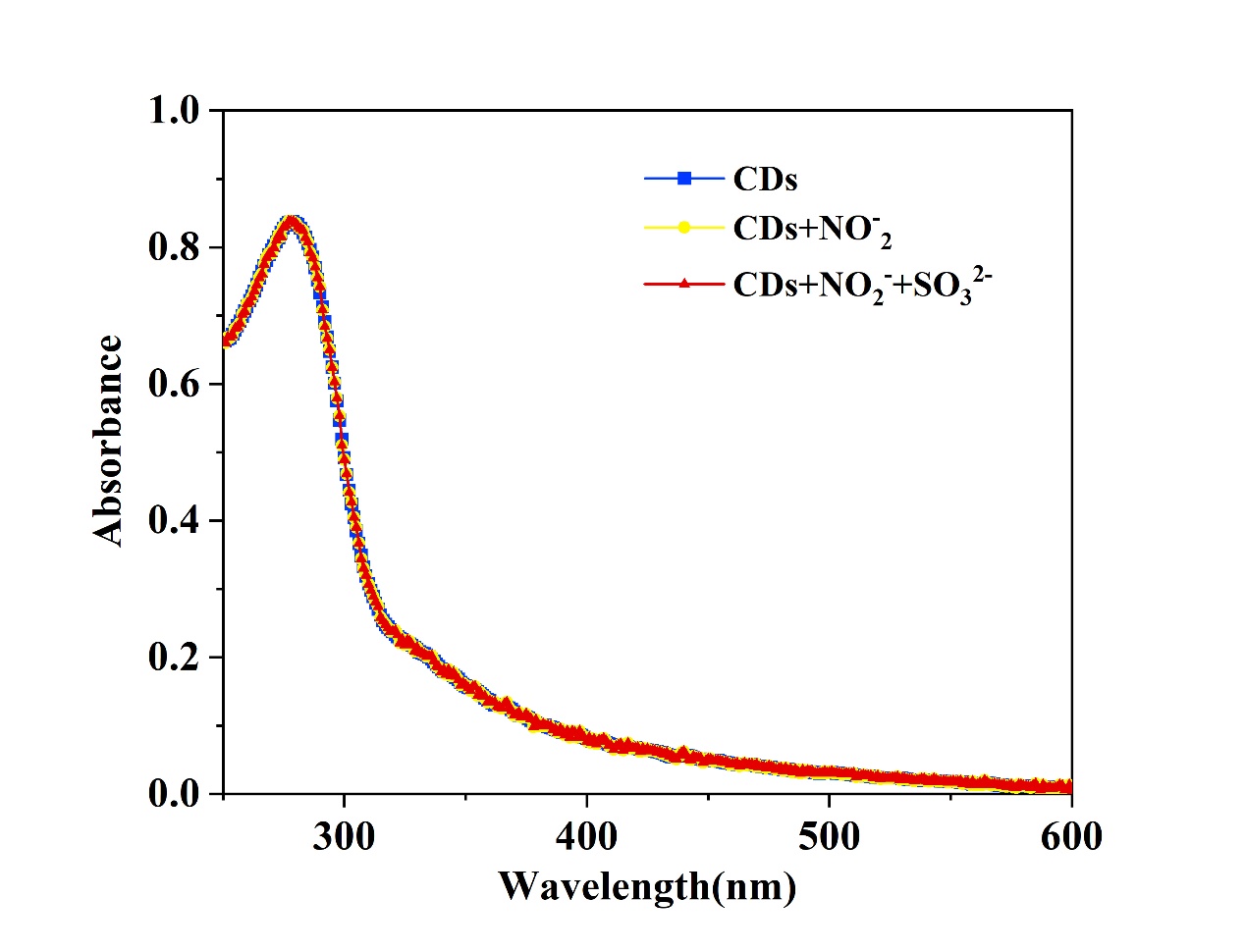


**Figure S3.** UV-spectra of CDs, CDs/NO_2_^-^ and CDs/NO_2_^-^/SO_3_^2-^.
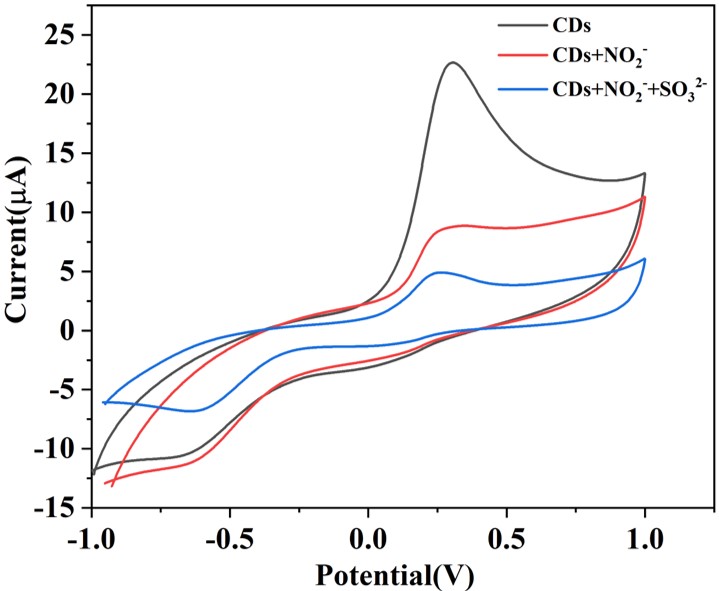


**Figure S4.** Cyclic voltammograms recorded on CDs, CDs/NO_2_^-^ and CDs/NO_2_^-^/SO_3_^2-^.


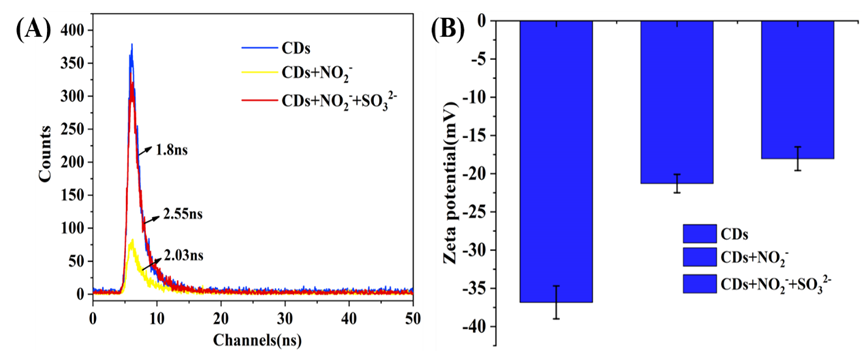


**Figure S5**. (A) Fluorescence lifetime of CDs, CDs/NO_2_^-^ and CDs/NO_2_^-^/SO_3_^2-^. (B) The surface potential of CDs, CDs/NO_2_^-^ and CDs/NO_2_^-^/SO_3_^2-^.

**
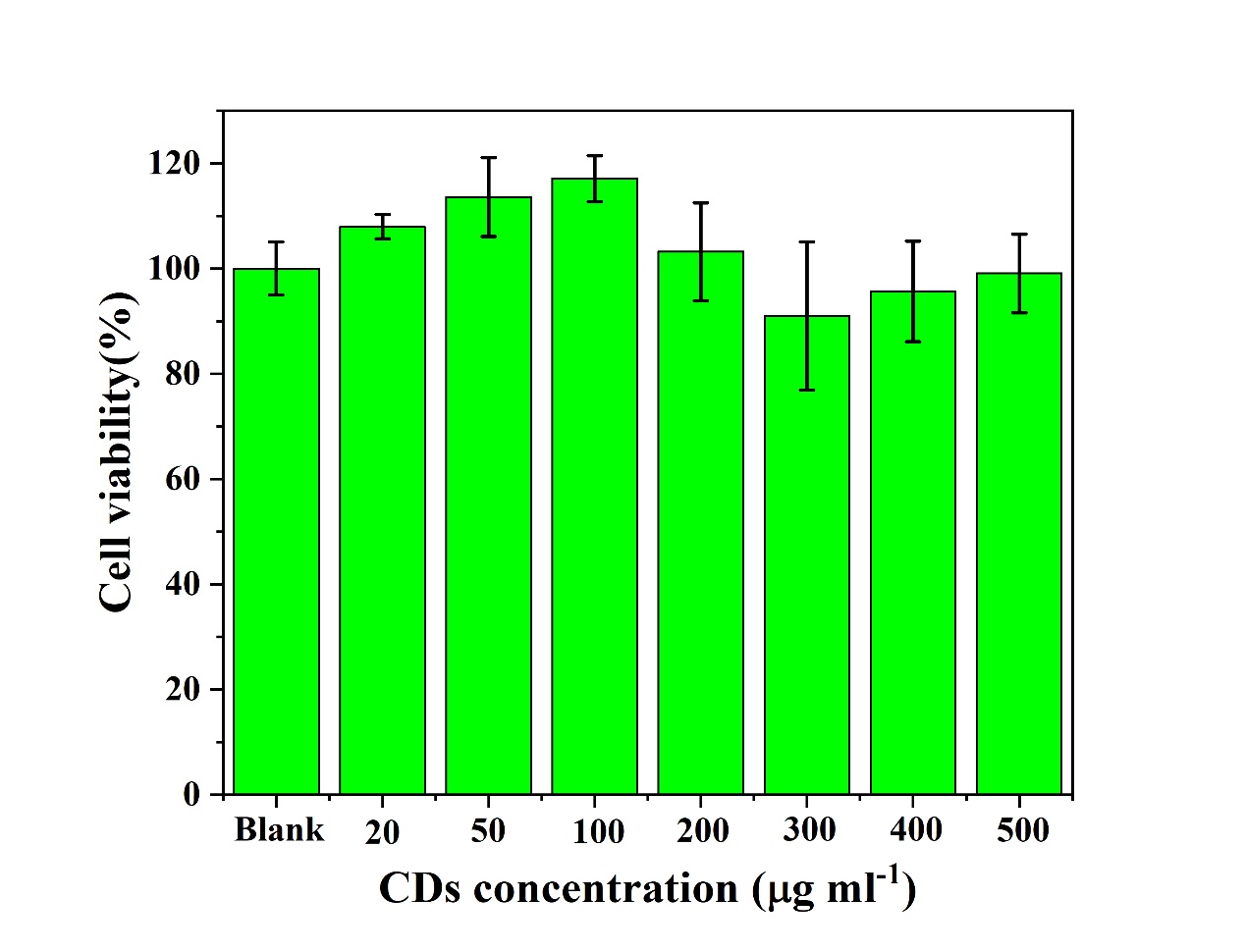
Figure S6.** Cell viability with different concentration of CDs for 24 h.

Table 2 The concentration of ions in cells and tissues.

| Ions | Concentration | Cells and tissues | References |
| --- | --- | --- | --- |
| Cu^2+^ | 10^-18^ **-** 10^-13^ M | Yeast cells and human blood plasma | (Tapiero et al., 2003) |
| Fe^3+^ | 12 **-** 28 μМ | Plasma | (Mojic et al., 2014) |
| Cys | 30 **-** 200 μМ | Intracellular | (Zhang et al., 2015) |
| GSH | 1 **-** 10 mM | Intracellular | (Zhang et al., 2015) |
| Ca^2+^ | 396 **±** 13 μМ | HeLa | (Tang et al., 2011) |
| Na^+^ | 12 mM | Intracellular | (Park et al., 2021) |
| K^+^ | 150 mM | Intracellular | (Park et al., 2021) |
| PO_4_^3-^ | 1.1 ± 0.2 mM | Plasma | (Furman et al., 1997) |

References:

Furman, S., Lichtstein, D., and Ilani, A. (1997). Sodium-dependent transport of phosphate in neuronal and related cells. Biochim Biophys Acta 1325(1), 34-40. doi: 10.1016/s0005-2736(96)00238-6.

Mojic, M., Bogdanovic Pristov, J., Maksimovic-Ivanic, D., Jones, D.R., Stanic, M., Mijatovic, S., et al. (2014). Extracellular iron diminishes anticancer effects of vitamin C: an in vitro study. Sci Rep 4, 5955. doi: 10.1038/srep05955.

Park, S.-H., Hwang, I., McNaughton, D.A., Kinross, A.J., Howe, E.N.W., He, Q., et al. (2021). Synthetic Na+/K+ exchangers promote apoptosis by disturbing cellular cation homeostasis. Chem. doi: 10.1016/j.chempr.2021.08.018.

Tang, S., Wong, H.C., Wang, Z.M., Huang, Y., Zou, J., Zhuo, Y., et al. (2011). Design and application of a class of sensors to monitor Ca2+ dynamics in high Ca2+ concentration cellular compartments. Proc Natl Acad Sci U S A 108(39), 16265-16270. doi: 10.1073/pnas.1103015108.

Tapiero, H., Townsend, D.M., and Tew, K.D. (2003). Trace elements in human physiology and pathology. Copper. Biomedicine & Pharmacotherapy 57(9), 386-398. doi: 10.1016/s0753-3322(03)00012-x.

Zhang, Y., Shao, X., Wang, Y., Pan, F., Kang, R., Peng, F., et al. (2015). Dual emission channels for sensitive discrimination of Cys/Hcy and GSH in plasma and cells. Chem Commun (Camb) 51(20), 4245-4248. doi: 10.1039/c4cc08687b.
